# PEEK-OPTIMA^™^ as an alternative to cobalt chrome in the femoral component of total knee replacement: A preliminary study

**DOI:** 10.1177/0954411916667410

**Published:** 2016-09-16

**Authors:** Raelene M Cowie, Adam Briscoe, John Fisher, Louise M Jennings

**Affiliations:** 1 Institute of Medical and Biological Engineering, University of Leeds, Leeds, UK; 2 Invibio Ltd, Technology Centre, Hillhouse International, Thornton Cleveleys, UK

**Keywords:** Joint simulators, knee prostheses, orthopaedic tribology, wear analysis/testing, biomaterials, PEEK-OPTIMA^™^, ultra-high-molecular-weight polyethylene

## Abstract

PEEK-OPTIMA^™^ (Invibio Ltd, UK) has been considered as an alternative joint arthroplasty bearing material due to its favourable mechanical properties and the biocompatibility of its wear debris. In this study, the potential to use injection moulded PEEK-OPTIMA^™^ as an alternative to cobalt chrome in the femoral component of a total knee replacement was investigated in terms of its wear performance. Experimental wear simulation of three cobalt chrome and three PEEK-OPTIMA^™^ femoral components articulating against all-polyethylene tibial components was carried out under two kinematic conditions: 3 million cycles under intermediate kinematics (maximum anterior-posterior displacement of 5 mm) followed by 3 million cycles under high kinematic conditions (anterior-posterior displacement 10 mm). The wear of the GUR1020 ultra-high-molecular-weight polyethylene tibial components was assessed by gravimetric analysis; for both material combinations under each kinematic condition, the mean wear rates were low, that is, below 5 mm^3^/million cycles. Specifically, under intermediate kinematic conditions, the wear rate of the ultra-high-molecular-weight polyethylene tibial components was 0.96 ± 2.26 mm^3^/million cycles and 2.44 ± 0.78 mm^3^/million cycle against cobalt chrome and PEEK-OPTIMA^™^ implants, respectively (p = 0.06); under high kinematic conditions, the wear rates were 2.23 ± 1.85 mm^3^/million cycles and 4.44 ± 2.35 mm^3^/million cycles, respectively (p = 0.03). Following wear simulation, scratches were apparent on the surface of the PEEK-OPTIMA^™^ femoral components. The surface topography of the femoral components was assessed using contacting profilometry and showed a statistically significant increase in measured surface roughness of the PEEK-OPTIMA^™^ femoral components compared to the cobalt chrome implants. However, this did not appear to influence the wear rate, which remained linear over the duration of the study. These preliminary findings showed that PEEK-OPTIMA^™^ gives promise as an alternative bearing material to cobalt chrome alloy in the femoral component of a total knee replacement with respect to wear performance.

## Introduction

Polyether ether ketone (PEEK) is a thermoplastic polymer which has been used clinically in the spine and investigated for use as a biomaterial in trauma and orthopaedics due to its favourable mechanical properties and relative bioinertness.^1,2^ There has been growing interest in its use as an arthroplasty bearing material either in its natural, unfilled form or reinforced with carbon fibres (CFR-PEEK). Natural PEEK has been used in the spine in PEEK-on-PEEK articulations, where pre-clinical studies have demonstrated an equivalent wear rate for PEEK cervical (NuNec)^3^ and lumbar disc replacements (NuBac) compared to conventional materials,^4^ and although clinical follow-up has been relatively short term, the implants have shown promise.^5^ CFR-PEEK has been considered for use as acetabular cups in total hip replacement, and experimental wear simulation under standard gait conditions has shown lower wear rates than cross-linked ultra-high-molecular-weight polyethylene (UHMWPE) against ceramic heads^6–8^ although a 5-year follow-up from clinical trials of the Mitch cup has yielded a revision rate of 4 in 25 due to loosening and squeaking.^9^ CFR-PEEK has exhibited low wear experimentally in the tibial component of a highly conforming unicompartmental knee replacement.^10^ However, despite promise from experimental wear simulation in low contact stress situations, in high contact stress environments, there are questions about the suitability of CFR-PEEK^11,12^ and PEEK^13^ and to date there are minimal clinical data.^14^

The material of interest in this study was unfilled PEEK-OPTIMA^™^ manufactured by Invibio Biomaterials Solutions Ltd (Thornton Cleveleys, UK)^1,15^ and injection moulded to a geometry for use as the femoral component in total knee replacement. There are several potential advantages of using PEEK over cobalt chrome in this application. For example, the lower stiffness of PEEK compared to cobalt chrome may reduce implant loosening caused by stress shielding and bone resorption.^16–18^ Also, when coupled with an all-polyethylene tibial component as proposed in this study, the implant will be metal free, which will be of particular benefit to patients with metal sensitivity.^19^

Wear debris induced osteolysis leading to aseptic loosening,^20,21^ however, remains one of the primary failure mechanisms of total knee replacements;^22^ therefore, there is a continuing interest in investigating novel material combinations for joint replacement. The wear performance of such novel material combinations should be assessed under a wide envelope of clinically relevant conditions to determine their efficacy, reliability and safety prior to implantation.^23^ With the use of implants in younger more active patients, the threshold for osteolysis^24,25^ is reached sooner and implant longevity diminishes. Hence, in this study, wear rates were investigated in a knee joint simulator under different kinematic conditions representative of different levels of patient activity.

The aim of this study was to assess the suitability of PEEK-OPTIMA^™^ for use as an alternative bearing material to cobalt chrome in the femoral component of total knee replacements in terms of its wear performance. It was hypothesised that the wear rate of the UHMWPE tibial components would be equivalent when articulating against cobalt chrome or PEEK-OPTIMA^™^ femoral components of similar initial surface topography and geometry.

## Materials and methods

**Figure 1.**
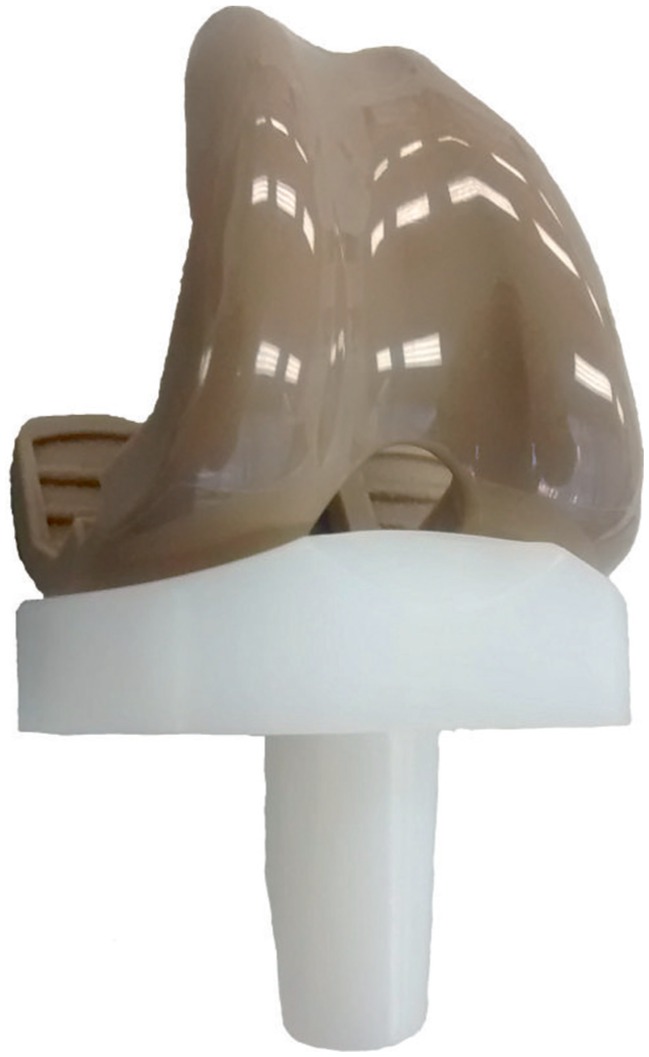
Injection moulded PEEK-OPTIMA^™^ femoral component coupled with an all-polyethylene tibial component.

Three injection moulded PEEK-OPTIMA^™^ femoral components (Invibio Knees Ltd, UK) with initial mean surface roughness (Ra) of 0.02 µm and three Co–Cr–Mo (cobalt chrome) femoral components (Ra = 0.02 µm) (Maxx Medical Pvt Ltd, PA, USA) were tested against GUR1020 (conventional, unsterilised) all-polyethylene tibial components (Figure 1) (Maxx Medical Pvt Ltd). The surface topography of the PEEK-OPTIMA^™^ femoral components was as-moulded; there was no additional post-processing of the articulating surfaces of the implants, and the geometry of the PEEK-OPTIMA^™^ implant was based on the engineering drawing of the cobalt chrome component.

All implants were right, mid-sized, cruciate retaining implants. Two additional UHMWPE tibial components were used as unloaded soak controls to compensate for the moisture uptake during the study.^26^ Prior to the start of testing, the UHMWPE components were soaked in sterile water for a minimum of 2 weeks to maximise their moisture uptake.

**Figure 2.**
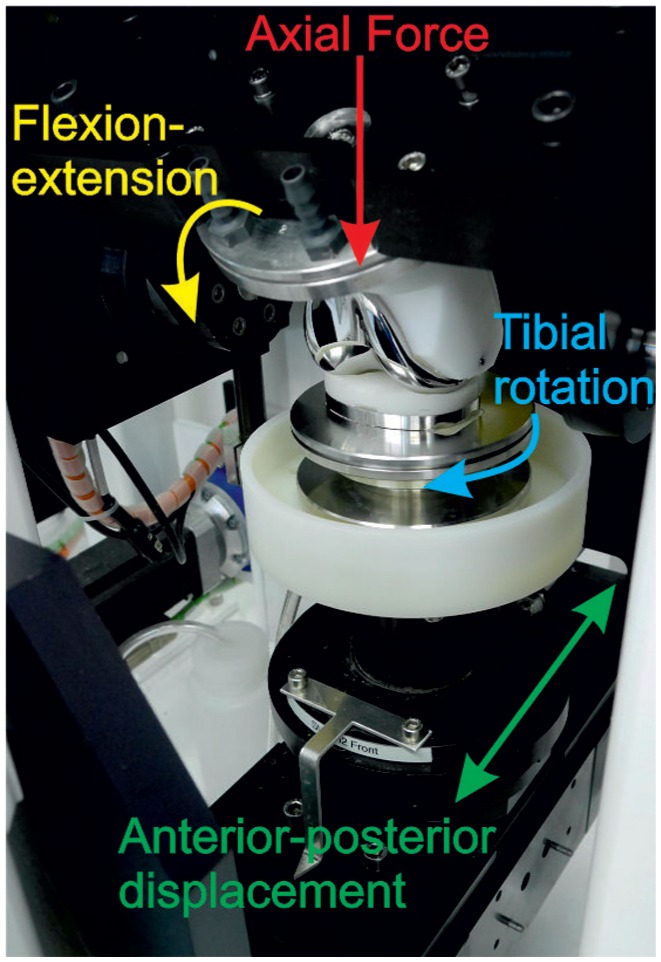
The four controlled axes of motion in a knee wear simulator.

**Figure 3.**
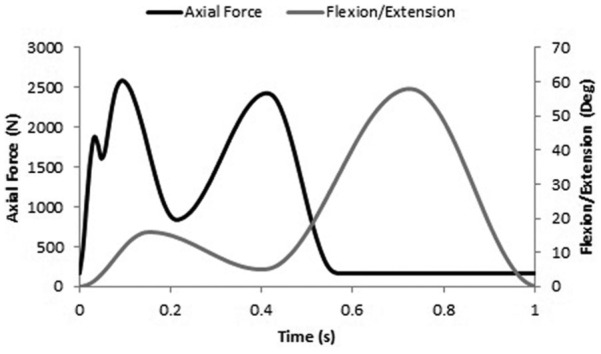
Axial force (AF) and flexion/extension (FE) input profiles.

**Figure 4.**
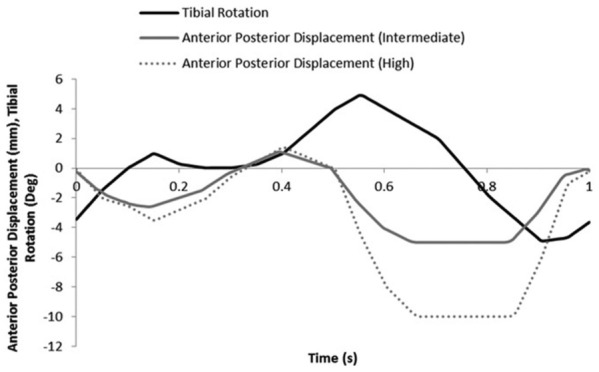
Tibial rotation (TR) and anterior-posterior displacement (AP) input profiles for intermediate and high kinematic conditions.

Experimental wear simulation was carried out on a six-station ProSim electro pneumatic knee simulator (Simulation Solutions, UK). Each station had 6 degrees of freedom with four controlled axes of motion as shown in Figure 2: axial force (AF), flexion/extension (FE), anterior-posterior (AP) displacement and tibial rotation (TR). The AF (maximum: ∼2800 N) and FE (0°–58°) were taken from the international standard for wear testing (ISO 14243-3) (Figure 3).^26^ The AP and TR were delivered through the tibial side of the implant and were displacement controlled. Displacement control was selected because prostheses do not have intrinsic constraint within the design and relied on soft tissue constraints in vivo.^27^ The TR was consistent for all tests and set at ±5°, and two AP displacement conditions were used. Intermediate kinematics applied an AP displacement of 0–5 mm, and under high kinematics, the AP displacement was larger, that is, 0–10 mm (Figure 4). The shape of the input profiles was based on the natural kinematics of the knee as described by Lafortune et al.^28^ The magnitude of the displacement under intermediate kinematics was similar to that described in the ISO standard,^26^ and under high kinematics, the magnitude of the displacement was based on gait analysis of the natural knee of healthy subjects.^28^ Abduction/adduction motion was passive and the AF was offset 7% of the width of the implant in a medial direction from the tibial axis as described in the ISO standard.^26^ The cycle frequency was 1 Hz.

The femoral components were set up on the distal centre of rotation to facilitate femoral rollback as per standard practice at Leeds^29^ with the tibial components cemented with respect to the position of the femoral components. The fixation of the tibial components was unique to each implant which minimised micro motion between the implant and the cement mantle, and the tibial components could be removed from the cement mantle for gravimetric analysis. The femoral and tibial components remained paired for the duration of the study but to reduce the interstation variation, each million cycles (MC), the implants were moved to the adjacent station. The tests were carried out in 25% (v/v) new born calf serum diluted with 0.03% (v/v) sodium azide solution to retard the bacterial growth giving a final protein concentration of 15 g/L. Approximately, for every 0.3 MC, the lubricant was replaced. The study was carried out at room temperature to minimise the potential artefacts due to protein deposition and denaturation at elevated temperature^30^ and to investigate the potential for frictional heating of the lubricant to occur in the all-polymer implant.

Prior to the start of the study, the simulator was calibrated and the tibial components were cleaned for 10 min in 70% propan-2-ol in an ultrasonic bath before drying in air and being left to stabilise in a temperature (20° ± 1°) and humidity (45% ± 5%) controlled environment for 48 h. Gravimetric analysis of the UHMWPE tibial components was carried out using a Mettler Toledo XP205 (Mettler Toledo, Leicester, UK) digital microbalance with a 0.01 mg resolution. Measurements were repeated until five consecutive measurements fell within a range of ±0.05 mg. The same cleaning, drying and weighing protocol was used at each gravimetric measurement point. Surface roughness measurements of the articulating surfaces were taken using a Taylor Hobson PGI800 contacting Form Talysurf (Taylor Hobson, Leicester, UK) with a 2 µm conical tip stylus. Filtering and cut-offs were used app-ropriate to the material and to ISO 4288:1996.^31^ The surface roughness parameters of interest were as follows: the mean surface roughness (Ra), the maximum profile height above the mean line (Rp) and the maximum profile depth below the mean line (Rv).

Three MC of wear simulation were carried out under intermediate kinematics, the bulk lubricant temperature was monitored daily, close to the articulating surfaces using a Fluke 51 II thermocouple (Fluke, Washington, USA) and the wear of the UHMWPE tibial components assessed at 1 and 3 MC. At the conclusion of the study under intermediate kinematics, the surface topography of the articulating surfaces was reassessed. The test was then resumed using the same components, but running a high kinematic profile with an increased AP displacement for an additional 3 MC. The wear of the UHMWPE tibial components was measured at 1 and 3 MC (minimum). The surface topography of the articulating surfaces was assessed at the completion of the study. Three sets of implants were tested for each material combination.

For each set of three knees and each set of kinematic conditions, the mean wear rate (mm^3^/MC), bulk lubricant temperature and Ra, Rp and Rv plus 95% confidence limits were calculated. The mean wear rate was calculated using linear regression. Statistical analysis was carried out using a student’s t-test,^32^ comparing the PEEK implants with the cobalt chrome implants at each time point with significance taken at p < 0.05.

The data associated with this article are openly available from the University of Leeds Data Repository.^33^

## Results

**Figure 5.**
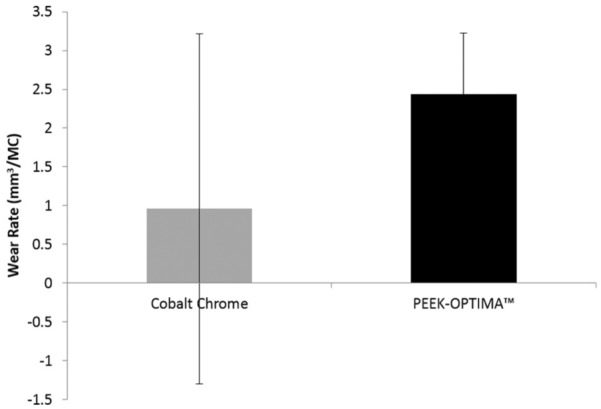
Mean wear rate (mm^3^/MC) with 95% confidence limits of UHMWPE tibial components against cobalt chrome and PEEK-OPTIMA^™^ femoral components under intermediate kinematic conditions (n = 3).

**Figure 6.**
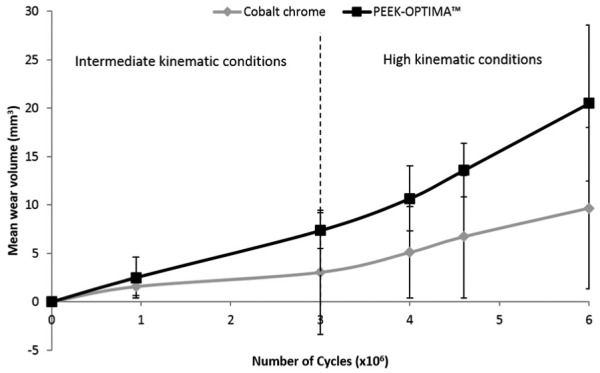
Mean wear volume (mm^3^) with 95% confidence limits of UHMWPE tibial components against cobalt chrome and PEEK-OPTIMA^™^ femoral components under intermediate and high kinematic conditions (n = 3).

Following 3 MC of intermediate kinematics, the wear rate of cobalt chrome-on-UHMWPE was 0.96 ±2.26 mm^3^/MC and the wear rate of PEEK-OPTIMA^™^ on-UHMWPE was 2.44 ± 0.78 mm^3^/MC (Figure 5). There was no significant difference in the wear of the UHMWPE tibial components articulating against the different materials (p = 0.06). After 3 MC of wear simulation under intermediate kinematics, a polished region was apparent in the contact area of the tibial components, the cobalt chrome implants had discrete scratches running in an AP direction on their surface and the PEEK-OPTIMA^™^ femoral components had a high density of light scratches where there had been contact between the two surfaces. Table 1 shows the surface topography of the articulating surfaces of the femoral components. Prior to the start of wear simulation, there was no significant difference (p > 0.05) between the measured Ra, Rp or Rv of the PEEK-OPTIMA^™^ or cobalt chrome femoral components. After 3 MC of wear simulation under intermediate kinematics, there was a significant difference (p < 0.05) in the Ra, Rp and Rv of the PEEK-OPTIMA^™^ femoral components compared to the cobalt chrome implants. After 3 MC wear simulation, the UHMWPE tibial components had a polished region in the wear area where the machining marks had been removed. For the tibial components articulating against the PEEK-OPTIMA^™^ femoral components, within the burnished region, light, linear scratching was apparent. As a result of this, the mean surface roughness (Ra) of the tibial components articulating against PEEK-OPTIMA^™^ was significantly (p < 0.05) higher than those articulating against cobalt chrome after 3 MC wear simulation under intermediate kinematic conditions (Table 2). Over the duration of the study, the wear rate was linear for both material combinations as shown in Figure 6. Under intermediate kinematics, the R^2^ value for the wear rate of the all-polymer knee was 0.99 and 0.95 for the conventional materials. The change in surface topography of the PEEK-OPTIMA^™^ femoral components did not appear to influence the wear rate. The mean bulk lubricant temperature in the all-polymer knee was 29.5 °C which was significantly (p = 0.01) higher than that of the conventional metal-on-UHMWPE implant (28.0 °C).

**Table 1.** Surface roughness measurements (mean ± 95% confidence limits) of cobalt chrome and PEEK-OPTIMA^™^ femoral components.

Parameter (µm)	Cobalt chrome femoral components		PEEK-OPTIMA^™^ femoral components	
	Pre-test	Post-test	Pre-test	Post-test
Ra	0.02 ± 0.00	0.03 ± 0.04	0.02 ± 0.01	0.23 ± 0.18
Rp	0.08 ± 0.00	0.10 ± 0.07	0.08 ± 0.01	0.52 ± 0.49
Rv	0.06 ± 0.01	0.09 ± 0.09	0.07 ± 0.01	1.29 ± 0.56

MC: million cycles.

Measurements taken in a medial-lateral direction prior to testing and following 3 MC wear simulation under intermediate kinematic conditions (n = 3).

**Table 2.** Mean surface roughness (±95% confidence limits) of UHMWPE tibial components articulating against PEEK-OPTIMA^™^ and cobalt chrome femoral components tested after 3 MC intermediate kinematic conditions and 3 MC high kinematic conditions (n = 3).

Parameter (µm)	UHMWPE tibial components articulating against cobalt chrome			UHMWPE tibial components articulating against PEEK-OPTIMA^™^		
	Pre-test	3 MC intermediate	3 MC high	Pre-test	3 MC intermediate	3 MC high
Ra	0.52 ± 0.11	0.30 ± 0.20	0.30 ± 0.07	0.49 ± 0.12	0.47 ± 0.06	0.67 ± 0.35
Rp	1.86 ± 0.30	0.94 ± 0.67	0.82 ± 0.29	1.80 ± 0.26	1.24 ± 0.45	1.91 ± 0.31
Rv	1.55 ± 0.26	1.13 ± 0.97	0.55 ± 0.19	1.45 ± 0.35	1.67 ± 0.80	0.93 ± 0.17

MC: million cycles.

**Figure 7.**
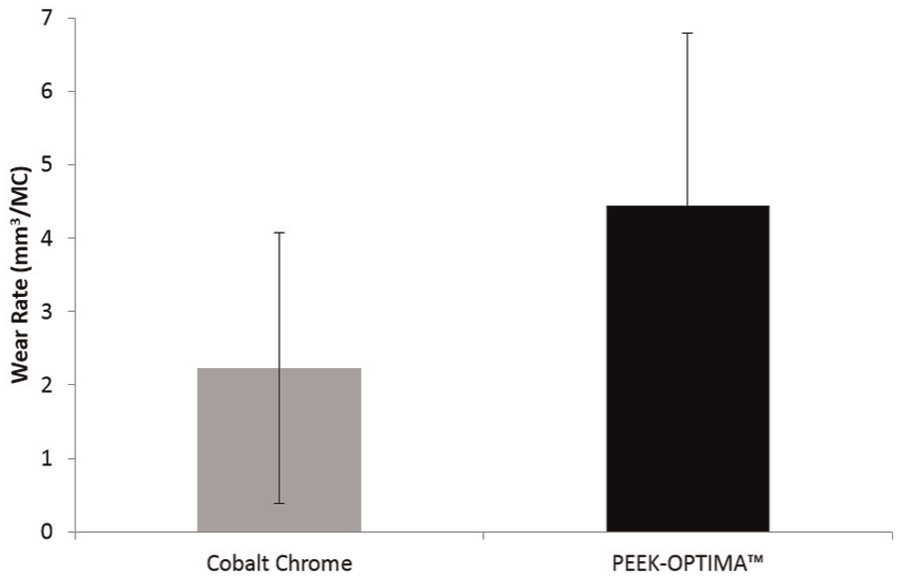
Mean wear rate (mm^3^/MC) with 95% confidence limits of UHMWPE tibial components against cobalt chrome and PEEK-OPTIMA^™^ femoral components under high kinematic conditions (n = 3).

The same implants were then tested for an additional 3 MC under high kinematic conditions with an increased AP displacement, reflecting a higher demand patient. The mean wear rate of the conventional implant materials as shown in Figure 7 was 2.23 ± 1.85 mm^3^/MC and the wear of the all-polymer knee was significantly higher than the conventional implant materials, p = 0.03 (4.44 ± 2.35 mm^3^/MC). The wear rate under high kinematic conditions remained linear over the duration of the study for both the all-polymer implant (R^2^ = 0.99) and the conventional metal-on-polyethylene implant (R^2^ = 0.99). Analysis of the surface of the femoral components (Table 3) showed a significant difference (p < 0.05) between the surface roughness parameters (Ra, Rp and Rv) of the PEEK and the cobalt chrome implants after 3 MC intermediate and 3 MC high kinematics. The scratches evident on the surface of the PEEK implants after 3 MC of wear simulation under intermediate kinematics were still visible but following an additional 3 MC under high kinematics; the measured values for Ra, Rv and Rp for the PEEK components were similar to those taken after 3 MC of intermediate kinematics and there was no apparent further deterioration of the surfaces. The surface roughness of the tibial components, however, was significantly higher (p < 0.05) for the implants articulating against PEEK-OPTIMA^™^ compared to those articulating against cobalt chrome (Table 2) for all the surface roughness parameters of interest. When tested under high kinematics, the mean bulk lubricant temperature of the all-polymer implant was significantly higher (29.7 °C) (p < 0.01) than the lubricant temperature measured in the conventional materials (27.6 °C).

**Table 3.** Surface roughness measurements (mean ± 95% confidence limits) of cobalt chrome and PEEK-OPTIMA^™^ femoral components.

Parameter (µm)	Cobalt chrome femoral components		PEEK-OPTIMA^™^ femoral components	
	Pre-test	Post-test	Pre-test	Post-test
Ra	0.03 ± 0.04	0.03 ± 0.01	0.23 ± 0.18	0.23 ± 0.16
Rp	0.10 ± 0.07	0.09 ± 0.03	0.52 ± 0.49	0.54 ± 0.38
Rv	0.09 ± 0.09	0.10 ± 0.04	1.29 ± 0.56	0.74 ± 0.43

MC: million cycles.

Measurements taken in a medial-lateral direction prior to testing and following 3 MC wear simulation under high kinematic conditions (n = 3).

## Discussion

The aim of this study was to assess the suitability of PEEK-OPTIMA^™^ for use as an alternative bearing material to cobalt chrome in the femoral component of total knee replacements in terms of its wear performance. The wear of the all-polymer implant was directly compared to that of a conventional metal-on-polyethylene implant of similar geometry and surface topography, and experimental wear simulation was carried out under different kinematic conditions indicative of different patient activity levels.

After 3 MC of experimental wear simulation under intermediate kinematic conditions, the wear performance of an all-polymer PEEK-OPTIMA^™^-on-UHMWPE total knee replacement was comparable to a conventional metal-on-UHMWPE implant of similar initial geometry and surface topography. To put these results into context, previous experimental wear simulation of fixed bearing knee replacements under similar intermediate kinematic conditions has shown wear rates of approximately 8.6 mm^3^/MC with stabilised UHMWPE^34^ and 2.6 mm^3^/MC for moderately cross-linked UHMWPE^35^ against cobalt chrome femoral components; in moderately cross-linked UHMWPE, the wear rate is considered to be low (<5 mm^3^/MC). Therefore, the wear rate of 0.96 ± 2.26 mm^3^/MC for the metal-on-UHMWPE implants in this study with a conventional UHMWPE tibial insert was also considered to be low, possibly due to their low conforming design.^34^ Measuring low wear rates of UHMWPE (<5 mm^3^/MC) by gravimetric analysis is difficult, and there is a loss of reliability in the measurement technique which makes the differentiation between the effect of variables being studied and uncontrolled and random errors in the system difficult. This, combined with the small sample size, may have contributed to the high variability in the measured wear rates of the tibial components.^20^ The low wear of the all-polymer knee was consistent with previous simple geometry wear simulation of PEEK-on-UHMWPE.^36^

Damage to the PEEK-OPTIMA^™^ femoral components was observed in the form of scratching parallel to the principal direction of sliding. Brown and Bao^3^ also reported the damage to the articulating surfaces of PEEK-on-PEEK cervical discs early in a spine simulator study, however; despite the initial change in surface topography, the wear rate remained constant as observed in our study. However, there was evidence that the linear scratching on the PEEK-OPTIMA^™^ femoral also caused scratching in the wear scar on the UHMWPE tibial component.

The bulk lubricant temperature was higher in the all-polymer knee than in the conventional implant; this elevated temperature could be attributed to frictional heating^37^ due to the anticipated higher friction in this material combination^8^ and poor dissipation of heat due to the low thermal conductivity of the polymers.^38^ Although higher friction bearing couples have exhibited frictional heating in vivo,^39^ the clinical relevance of the elevated temperatures measured in our tests is unknown. The continuous running of the simulator may have accentuated the frictional heating^40^ and led to a test artefact^41^ by creating differing environmental test conditions for the different materials. The lubricant used was 25% serum analogous to synovial fluid with the final protein concentration (15 g/L) matched to that in vivo,^42^ and tests were carried out at room temperature to minimise the test artefacts caused by denaturation of the protein-based lubricant. However, to minimise the influence of frictional heating, rest periods could have been incorporated into the test protocol.

Having demonstrated a similar rate of wear of UHMWPE against the two femoral materials under intermediate kinematics, the wear of the same implants under high kinematic conditions with an increase in the AP displacement was investigated. Using the same implants for both kinematic conditions, the potential for variability in set up of the implants has been minimised, the study has started to investigate the influence of long-term testing on the wear of the PEEK-OPTIMA^™^ implant and the study is more representative of changes in patient’s gait as they perform different activities. Typical wear rates for fixed bearing knees under high kinematic conditions tested on the same simulator as in this study were 15.9 mm^3^/MC^34^ for stabilised UHMWPE and 6.7 mm^3^/MC for moderately cross-linked UHMWPE.^35^ It was anticipated that the change in surface topography of the PEEK-OPTIMA^™^ femoral component would influence the wear rate of the UHMWPE tibials; however, the wear rate remained low (<5 mm^3^/MC) and was linear over the duration of the study, likely due to the orientation of the scratches in the principal direction of sliding. Surface topography measurements of the PEEK-OPTIMA^™^ femoral components following 3 MC of high kinematics showed no further change to their surface compared to measurements taken after 3 MC of intermediate kinematics. However, the wear rate of the PEEK-on-UHMWPE was statistically significantly higher than metal-on-UHMWPE under these conditions. It was a limitation of this study that the tests under the different kinematic conditions were not independent since the same samples were tested first under intermediate kinematics before testing under high kinematics. Therefore, it is possible that changes in the surface topography of the femoral components as a result of the intermediate kinematic conditions test may have influenced the wear under high kinematics. However, this appears not to be the case since the wear rate under both the intermediate and high kinematic conditions remained linear over the duration of the study for both the all-polymer implant and the conventional metal-on-polyethylene implant. Long-term testing with a larger set of samples will be necessary to fully assess whether the changes in surface topography of the PEEK-OPTIMA^™^ femoral component influence the wear rate of UHMWPE tibial components.

This was a preliminary study focusing solely on the wear performance of the all-polymer knee implant and therefore there were several other limitations, such as sample size. Three sets of implants were studied for each material combination, restricted by the number of stations in the simulator and the necessity to carry out control tests of conventional implants of similar geometry in parallel. This is the best practice and allows the influence of the different femoral materials on UHMWPE wear to be directly compared. However, a larger sample size may have reduced the 95% confidence limits, making the statistical analysis more robust and giving greater evidence on which to draw conclusions. Another limitation was the use of unsterilised components. However, the proposed sterilisation route of the UHMWPE by ethylene oxide has been shown not to influence the mechanical properties or induce cross-linking and therefore the wear performance of the UHMWPE is not anticipated to be influenced by such sterilisation.^6,43^ In this study, the wear of the UHMWPE tibial components was assessed. Previous work on metal-on-polyethylene knees assumes all wear generated is from the UHMWPE. It is not known whether there was wear of the PEEK-OPTIMA^™^ femoral component as the implants could not be assessed by gravimetric analysis nor was a method available to assess potential wear geometrically. Future work will assess the wear debris generated by the all-polymer knee implant and compare its morphology and size distribution to that generated by a conventional metal-on-polyethylene implant. Furthermore, the tests conducted in this study were relatively short term, and longer duration simulation will be necessary to fully assess the long-term wear performance of the implant.

In conclusion, under intermediate kinematic conditions, the wear rate of the UHMWPE tibial components was independent of the femoral material as a similar rate of wear was shown against cobalt chrome and PEEK-OPTIMA^™^ femoral components of similar geometry. Under higher demand kinematics, the wear of the UHMWPE was significantly higher against PEEK than cobalt chrome, but the magnitude of the wear was considered to be low (<5 mm^3^/MC) against both materials, and measuring low rates of wear gives potential for measurement errors especially in a low sample size. Over the duration of this study, the surface of the PEEK-OPTIMA^™^ femoral components did change but this did not influence the wear rate in this short-term study. This study showed that PEEK-OPTIMA^™^ has potential for use as an alternative bearing material to cobalt chrome in total knee replacement; however, the study should be considered as generation of baseline data prior to further and long-term pre-clinical testing under a wider envelope of more adverse and clinically relevant conditions.
